# Lifetime suicidal thoughts, attempts, and lethality of attempts as major outcome domains of psychotic disorders: a 21-year prospective cohort study after a first-episode psychosis

**DOI:** 10.1017/S0033291725000443

**Published:** 2025-03-04

**Authors:** Victor Peralta, Lucía Moreno-Izco, Elena García de Jalón, Ana M. Sánchez-Torres, David Peralta, Lucía Janda, Manuel J. Cuesta, SEGPEPs group, X. Ansorena, A. Ballesteros, J. Chato, L. Fañanás, G. Gil-Berrozpe, E. Giné-Servé, R. Lorente, S. Papiol, M. Ribeiro, E. Rosado, A. Rosero

**Affiliations:** 1 Mental Health Department, Servicio Navarro de Salud, Pamplona, Spain; 2 Instituto de Investigación Sanitaria de Navarra (IdiSNA), Pamplona, Spain; 3 Department of Psychiatry, Hospital Universitario de Navarra, Pamplona, Spain; 4 Departamento de Ciencias de la Salud, Universidad Pública de Navarra (UPNA), Pamplona, España; 5 Department of Evolutionary Biology, Ecology and Environmental Sciences, Faculty of Biology, University of Barcelona, Biomedicine Institute of the University of Barcelona (IBUB), Barcelona, Spain; 6 Network Centre for Biomedical Research in Mental Health (CIBER of Mental Health, CIBERSAM), Spain; 7 Institute of Psychiatric Phenomics and Genomics (IPPG), University Hospital, LMU Munich, Munich, 80336, Germany; 8 Department of Psychiatry and Psychotherapy, University Hospital, LMU Munich, Munich, 80336, Germany

**Keywords:** first-episode psychosis, long-term follow-up, suicidal behavior, suicidal thoughts, suicide

## Abstract

**Background:**

Suicidal thoughts and behaviors (STBs) are a major concern in people with psychotic disorders. There is a need to examine their prevalence over long-term follow-up after first-episode psychosis (FEP) and determine their early predictors.

**Methods:**

Of 510 participants with FEP evaluated on 26 risk factors for later outcomes, 260 were reassessed after 21 years of follow-up for lifetime ratings of most severe suicidal ideation, number of suicide attempts, and lethality of the most severe attempt. Risk factors and STB outcomes were modeled using hierarchical linear regression analysis.

**Results:**

Over the 21-year follow-up period, 62.7% of participants experienced suicidal thoughts, 40.8% attempted suicide, and 18 died of suicide (3.5% case fatality and 20.6% proportionate mortality). Suicidal ideation was independently predicted by parental socioeconomic status, familial load of major depression, neurodevelopmental delay, poor adolescence social networks, and suicidal thoughts/behavior at FEP. The number of suicide attempts was independently predicted by years of follow-up, familial load of major depression, obstetric complications, childhood adversity, and suicidal thoughts/behavior at FEP. Lethality was independently predicted by familial load of major depression, obstetric complications, neurodevelopmental delay, and poor adolescence social networks. The proportion of variance in suicidal ideation, attempts, and lethality explained by the independent predictors was 29.3%, 21.2%, and 18.1%, respectively.

**Conclusions:**

STBs are highly prevalent in psychotic disorders and leads to substantial morbidity and mortality. They were predicted by a number of early risk factors, whose clinical recognition should contribute to improved prediction and prevention in people with psychotic disorders.

## Introduction

Suicidal thoughts and behaviors (STBs), including suicidal ideation, suicide attempts, and suicide death, are a major public health problem that puts a heavy burden on individuals, families, and society. Suicide is among the leading causes of death worldwide and more than 700 000 people die by suicide every year (World Health Organization, 2021). The reduction of suicide mortality has been prioritized by the WHO as a global target, and among the evidence-based interventions cited by the WHO to prevent suicide are ‘early identification, assessment, management, and follow-up of anyone who is affected by suicidal behaviours’ (World Health Organization, 2021).

The risk of suicide appears to be particularly elevated in people with schizophrenia and other psychotic disorders, numerous studies demonstrating an up to 20-fold increase in the relative risk of suicide in this diagnostic group compared with the general population (Nordentoft, Madsen, & Fedyszyn, 2015). Approximately 5% of people diagnosed with schizophrenia or psychosis die by suicide (Hor & Taylor, 2010; M. Nordentoft, Mortensen, & Pedersen, 2011; Palmer, Pankratz, & Bostwick, 2005; Starzer et al., 2023). This estimated rate remains unacceptably high, representing the largest single cause of excess mortality in people with schizophrenia (Correll et al., 2022) and contributing to a weighted average of 14.5 years of potential life lost (Hjorthøj, Stürup, McGrath, & Nordentoft, 2017). Given the magnitude of the problem, a better understanding of the factors associated with STBs in psychotic disorders is required to improve the assessment of suicidal risk and develop effective interventions.

The prediction of STBs continues to be a complex and unresolved question, both in nonclinical and clinical populations. A meta-analysis of 365 studies from the past 50 years concluded that prediction was only slightly better than the chance for suicidality outcomes, which was also evident in people with psychotic disorders (Franklin et al., 2017). While it is well known that suicide risk is highly influenced by contextual factors, little is known about the early risk factors for STBs. Indeed, the lack of progress in predicting STBs may be attributable to research predominantly focusing on isolated or proximal risk factors rather than trait vulnerabilities preceding the onset of mental disorders (Vidal-Ribas et al., 2022).

Despite extensive literature on the predictors of STBs after first-episode psychosis (FEP), which has been summarized in several systematic reviews and meta-analyses (Challis, Nielssen, Harris, & Large, 2013; Coentre, Talina, Góis, & Figueira, 2017; Huang, Fox, Ribeiro, & Franklin, 2018; Pompili et al., 2011; Sicotte, Iyer, Kiepura, & Abdel-Baki, 2021), there is poor agreement regarding the early predictors of STBs. To date, the most comprehensive systematic review in representative samples of individuals with FEP included 17 longitudinal studies (n = 14.907) with a mean follow-up length of 4.9 years (Sicotte et al., 2021). This review concluded that up to 27% of the participants had suicidal ideation, 21.6% had made at least one suicide attempt, 1–4.3% had died by suicide during follow-up; and only male sex, depressive symptoms, and suicide attempts occurring early during follow-up were associated with subsequent STBs. Other risk factors yielded conflicting results or were not associated with STBs; no study assessed factors associated specifically with suicidal ideation, and the heterogeneity of the individual studies precluded a meta-analysis.

The limitations of most previous studies include the relatively short length of follow-up, restricted set of predictors examined, and failure to control for potential confounders or among risk factors, the latter being particularly relevant as predictors of STBs are often intercorrelated (Franklin et al., 2017). Additionally, many studies erroneously group suicidal ideation and suicide attempt into a single suicidality outcome, which fails to distinguish factors associated with ideation from those associated with suicide attempts and death (Bakst, Rabinowitz, & Bromet, 2010; Nock et al., 2018).

To contribute to this important field, this study aimed to investigate the lifetime prevalence of STBs and their early risk factors using data from a large cohort of people with FEP, who were followed up for a mean of 21 years. We examined four STBs: lifetime most severe suicidal ideation, number of suicide attempts, lethality of the most severe attempt, and death by suicide. Self-harming behaviors were excluded from the study because they are behaviors carried out without any intention of suicide. Our specific aims were to (1) document the prevalence of STBs over the follow-up period; (2) estimate the potential predictive value of 26 early biopsychosocial risks and protective factors for suicidal ideation, number of suicide attempts, and lethality of the most severe attempt; and (3) examine the timing and mortality rates due to suicide over the follow-up.

## Methods

### Study design and population

**Figure 1.**
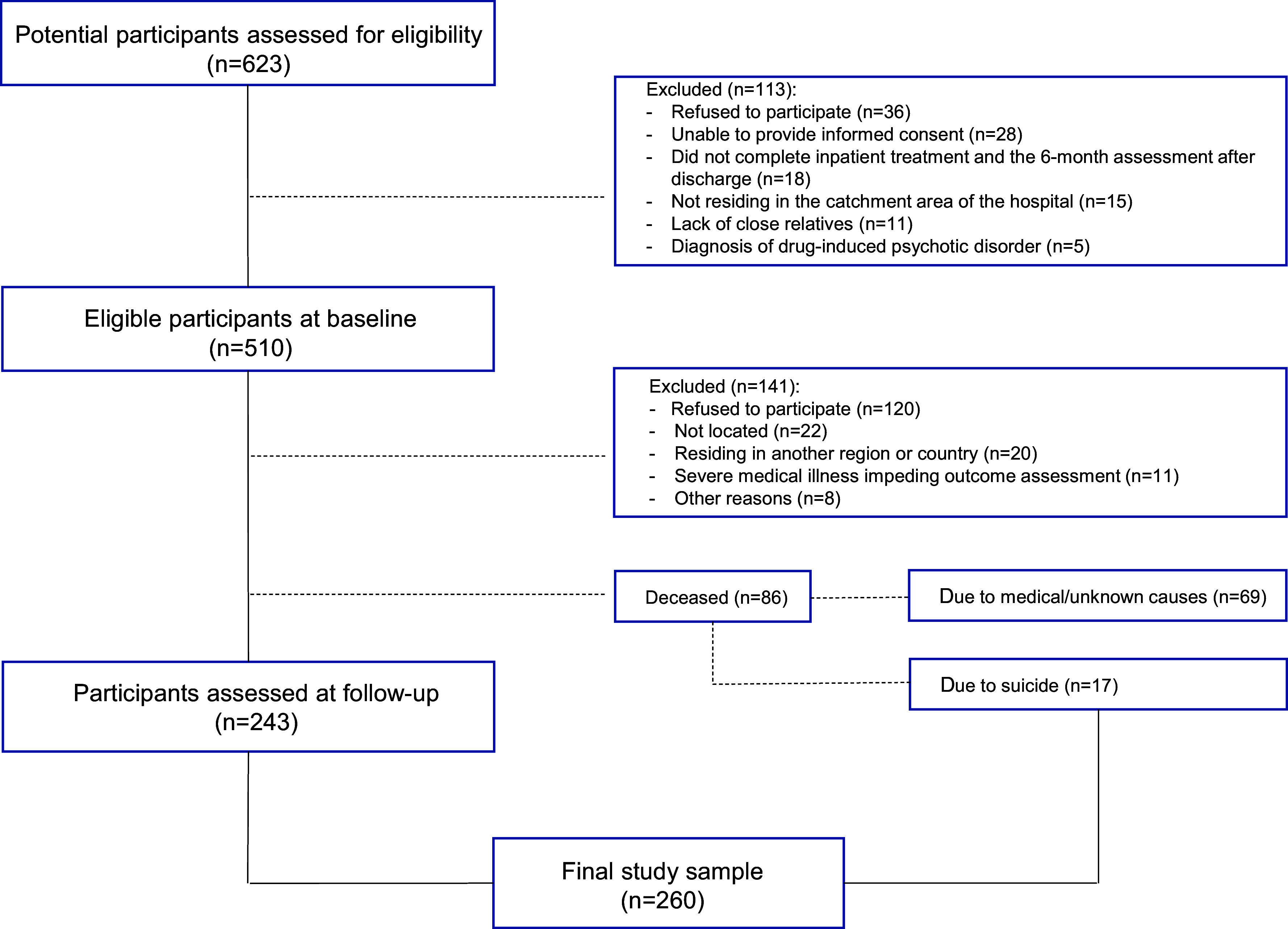
Flow diagram of included and excluded participants. *Note:* One participant who completed the follow-up assessments died by suicide shortly after the assessment; thus, the total number of deceased people at follow-up was 87 and the total number of suicide deaths was 18.

This was a longitudinal and naturalistic study of subjects with epidemiologically defined FEP. Eligible subjects were consecutive admitted to a psychiatric ward in Pamplona, Spain, which serves a defined catchment area for approximately 200 000 inhabitants, between January 1990 and December 2008 (Figure 1).

The baseline study cohort comprised subjects who met the following inclusion criteria: (a) admission for FEP fulfilling the DSM-III-R or DSM-IV criteria for a functional psychotic disorder; (b) age 15–65 years; (c) residence in the catchment area of the hospital; (d) completion of the inpatient treatment period; (e) close relatives available to provide broad background information; and (f) written informed consent. The exclusion criteria were as follows: (a) previous antipsychotic treatment for more than 2 months, (b) diagnosis of drug-induced psychosis, (c) history of serious medical or neurological disease, and (d) mental disability (IQ less than 70). The study methods are described in detail elsewhere (Peralta et al., 2022, 2021). Briefly, the main instrument for assessing clinical and diagnostic variables was the Comprehensive Assessment of Symptoms and History (CASH) (Andreasen, 1987, 1992). Regarding, the changing DSM criteria for psychotic disorders, a major advantage of the CASH is that it provides broad descriptive coverage for diagnosis using a variety of criteria from DSM-III to DSM-5. More specifically, the successive DSM editions defined virtually the same population of psychotic disorders, as shown by a kappa coefficient of >.95.

### Tracing and recontact procedures for the follow-up

Between January 2018 and May 2021, on average 21.1 years (SD = 5.59) after the baseline assessment, we sought to trace and reinterview the subjects to assess their clinical course and different outcomes of psychotic illness. We began by identifying deceased subjects, and then proceeded to locate the living subjects by postal mail and telephone. Subjects who did not respond to the first contact attempt were contacted again 2 months later. Finally, for the identified individuals who did not respond, we sought to contact and invite them via their treating psychiatrist or general practitioner. If subjects expressed an interest in the study, they were invited to meet with the field researchers to learn about and discuss participation.

In Spain, free universal healthcare is available for all citizens, and every citizen is assigned a unique identification number in the civil registration system. Because of this number, full data linkage of the participants in the cohort with health registers was possible. We extracted data from the following registers: (1) ‘Servicio Navarro de Salud’, (2) ‘Instituto Nacional de Estadística’, and (3) ‘Instituto Navarro de Medicina Legal y Forense’. We identified deceased subjects via electronic health records, such as the time and cause of death. To confirm those subjects died by suicide, we cross-checked the death records with the official forensic register. In Spain, all suspected suicides are investigated by a medical examiner with the power to order an autopsy and gather all the information necessary to reach a judgement on the cause of death.

All living participants and their legal representatives, if appropriate, signed written informed consent, and the local ethics committee granted ethical approval, including permission to access the clinical and official registers of deceased subjects. The authors assert that all procedures contributing to this work comply with the ethical standards of the relevant national and institutional committees on human experimentation and the Helsinki Declaration of 1975, as revised in 2008.

### Assessment of baseline predictors

A senior author (VP or MJC) assessed participants at baseline. We selected 26 baseline candidate risk factors of potential relevance for predicting suicidality outcomes, most of which have been examined in one or more previous studies of STBs in subjects with FEP. Risk factors were chronologically segmented according to their time from FEP into the following consecutive clusters: sociodemographics, familial risk, early developmental antecedents, late developmental antecedents, precipitating factors, illness-onset features, and FEP characteristics.

Sociodemographic variables were assessed using the CASH and included age, sex, length of follow-up, and five-level parental socioeconomic status (SES).

The familial risk of schizophrenia spectrum disorders, bipolar disorder, and major depressive disorder (MDD) was assessed in the participants’ first-degree relatives using the Family History-Research Diagnostic Criteria (FH-RDC) (Andreasen, Endicott, Spitzer, & Winokur, 1977). We estimated the familial load score for these disorders by considering family size and age structure according to Verdoux et al. (1996).

Early developmental antecedents included obstetric complications, which were assessed using the Lewis and Murray scale (Lewis, Owen, & Murray, 1989) and neurodevelopmental delay, which was assessed according to the scale published by (Shapiro et al., 1990), which rates developmental milestone attainment up to the age of three. The scale includes six specific milestones attained at expected age: sitting, standing, walking, talking words, talking sentences, and urine/feces control. A neurodevelopmental score was derived ranging from 0 (all milestones attained at expected age) to 6 (none of the milestones attained at expected age).

Late developmental antecedents included childhood adversity, which was assessed using the Global Family Environment Scale (Peralta et al., 2024; Rey et al., 1997), which indexes the global quality of the environment in which the child was raised up to age 12; adolescence psychosocial adjustment, which was rated by the CASH; and social networks during adolescence, which were assessed using the Social Support Scale (Surtees, 1980). Premorbid cognitive reserve was estimated according to established measures of premorbid intelligence, educational level, and leisure activities (Amoretti et al., 2020; Barnett, Salmond, Jones, & Sahakian, 2006) (see Supplementary Material).

Precipitating factors occurring within the 6 months before illness onset included psychosocial stressors rated per the DSM-III Axis IV (APA, 1980) as the overall severity of a stressor judged to have been a significant contributor to the development of disorder from 1 (no apparent stressor) to 7 (catastrophic) and the severity of substance abuse or dependence as rated per the CASH.

First-episode characteristics were psychopathology, as rated by the current state module of the CASH, which includes the Scale for the Assessment of Positive Symptoms (SAPS), the Scale for the Assessment of Negative Symptoms (SANS), global severity ratings for mania and depressive symptoms, and a severity rating for suicidal thoughts/attempts. Lack of insight was assessed using a specific item from the Positive and Negative Syndrome Scale (Peralta & Cuesta, 1994). Finally, illness severity and response to treatment were recorded using the corresponding sections of the Clinical Global Impression (CGI) scale (Guy, 1976).

### Assessment of STB outcomes

The lifetime version of the Columbia-Suicide Severity Rating Scale (CSSRS) (Posner et al., 2008) was used to assess three STBs in surviving participants: lifetime most severe suicidal ideation, total number of actual suicide attempts, and lethality or medical damage of the most serious attempt. Suicidal ideation and lethality were rated on a 6-point Likert scale (see Supplementary Material for a more extensive description of the CSSRS). The instrument was administered by two trained follow-up field interviewers (LMI and EGJ). They were blinded to the baseline characteristics of each subject and their background information and assessed the participants’ STBs based on information elicited during interviews with the participants, interviews with significant others, and medical records. Each participant who died by suicide was given the most severe score (5) on both the most severe suicidal ideation subscale (active suicidal ideation with a specific plan and intent) and the actual lethality of the most severe attempt subscale (death). Furthermore, for suicidal participants, the total number of suicide attempts and the last known DSM-5 diagnosis were obtained from the medical records.

Following Dutta et al. (Dutta et al., 2010), we defined proportionate mortality as the percentage of those who died within the follow-up period who committed suicide, and we defined case fatality as the percentage of the original cohort who died of suicide.

### Statistical analyses

Descriptive analyses and comparisons of patient characteristics were performed using the independent chi-square test or *t*-test, as appropriate. Continuous variables were examined for normality and transformed, as appropriate.

Pearson’s correlation coefficients were calculated between putative predictor variables and the three suicidality outcomes. These bivariate analyses formed the basis for multivariable hierarchical linear regression models that were constructed to estimate the unique contribution of the predictive variables to each suicidality outcome. The statistically significant predictor variables in the bivariate analyses were entered into the regression model one at a time in chronological order, as described above, and a final regression model was made from the independent variables that remained significant. The basic assumptions of the regression models were checked by examining residual plots, tolerance, and variance inflation factor, none of which were violated. Standardized β-values (and 95% CIs) were provided to allow comparisons between models. We estimated the incremental fit (∆R^2^) of the significant variables remaining in the model as an index of the unique amount of variance explained by the predictive variables in the outcome variance.

Kaplan–Meier analysis was performed to estimate the time-to-event (suicide) distribution over the follow-up period. We calculated life expectancy as the median survival time from baseline, which was the time at which half of the suicides occurred.

Power analysis was computed for the required sample size, given α = 0.05, power = 0.80, and a medium effect size. A significance level of 5% (two-tailed) was used for all analyses, which were performed using SPSS (version 21.0; SPSS, Chicago, Illinois, USA).

## Results

### Study sample

Of the 510 patients assessed at baseline, 243 were successfully followed-up (46.4% of the initial sample and 57.3% of the survivors) and completed the follow-up assessments. During the follow-up, a total of 17 participants died of suicide, which, together with those who completed the follow-up assessments, made the final study sample (n = 260, 51% of the initial sample) (Figure 1). One participant who completed the follow-up assessment committed suicide 4 months later; thus, the deceased subjects totaled 87, of whom 18 died of suicide.

A comparison of those with (n = 260) and without follow-up STBs information (n = 250) found that those with information were significantly younger (*p* < .001), which was due to the higher mean age of those excluded subjects due to severe medical illness (40.0, SD = 15.6) and nonsuicide mortality (40.7, SD = 14.5). No significant differences were found in any of the other baseline variables (Table 1).

**Table 1.** Baseline sociodemographic and clinical characteristics of cohort members included in the study (n = 260) and those excluded (n = 250)

	Included	Excluded	χ^2^ or t_(df)_	p
Sex, male, n (%)	151 (58.1)	153 (61.2)	0.616_(1)_	0.472
Age at study entry, y	27.6 (9.70)	32.0 (12.9)	4.337_(461,8)_	<0.001
Ethnic origin (Caucasian), n (%)	252 (51.9)	234 (48.1)	3.138_(1)_	0.076
Socioeconomic status score (1–5)	3.07 (0.72)	3.16 (0.67)	1.466_(508)_	0.143
Married/cohabiting at illness onset, n (%)	73 (30.0)	94 (35.2)	1.541_(1)_	0.214
Education, years	11.1 (3.38)	10.7 (3.44)	1.605_(508)_	0.109
Premorbid adjustment total score	5.37 (4.22)	5.58 (3.71)	0.594_(508)_	0.553
DUP, months	14.7 (34.3)	21.2 (45.6)	1.797_(462.2)_	0.071
Drug abuse, n (%)	87 (33.5)	94 (37.6)	0.953_(1)_	0.329
Type of onset (1 = acute, 4 = chronic)	2.58 (1.22)	2.74 (1.19)	1.522_(508)_	0.129
Compulsory admission, n (%)	81 (31.2)	82 (32.8)	0.159_(1)_	0.690
Antipsychotic drug-naïve status, n (%)	205 (78.8)	188 (75.2)	0.958_(1)_	0.328
Diagnosis, n (%):				
Schizophrenia	72 (29.6)	89 (33.3)	2.262_(7)_	0.944
Schizophreniform disorder	40 (16.5)	39 (14.6)		
Brief psychotic disorder	41 (16.9)	40 (15.0)		
Delusional disorder	16 (6.6)	23 (8.6)		
Schizoaffective disorder	13 (5.3)	12 (4.5)		
Mania/bipolar disorder	20 (8.2)	23 (8.6)		
Major depressive disorder	29 (11.9)	30 (11.2)		
Psychotic disorder NOS	12 (4.9)	11 (4.1)		
CGI, illness severity	2.70 (1.73)	2.84 (1.79)	0.872_(508)_	0.383
SAPS	9.48 (4.12)	9.01 (4.05)	1.662_(508)_	0.097
SANS	4.93 (5.21)	5.56 (5.38)	1.349_(508)_	0.178
Mood symptoms	1.85 (1.78)	1.66 (1.85)	1.229_(508)_	0.220
Suicidal thoughts/attempts	0.67 (1.29)	0.64 (1.22)	0.286_(508)_	0.775
CGI, efficacy index	1.56 (0.78)	1.66 (0.91)	1.355_(488.6)_	0.176

Abbreviations: DUP = Duration of Untreated Psychosis; NOS = Not Otherwise Specified; SAPS = Scale for the Assessment of Positive Symptoms; SANS = Scale for the Assessment of Negative Symptoms; CGI = Clinical Global Impression.

Compared to living participants, those who died by suicide had more suicide attempts (3.17 versus 1.21, *p* = 0.009) and a predominance of males (77.8% versus 56.6%, *p* = 0.069). The groups did not differ in the diagnosis of specific psychotic disorders (Supplementary Table S1).

### Prevalence of STBs

The distribution prevalence of the lifetime most severe suicidal ideation, total number of suicide attempts, and lethality of the most serious attempt are presented in Supplementary Tables S3 to S5. The lifetime rate of suicidal ideation was 62.7% (n = 163/260), and among these subjects, 55.2% (90/163) had suicidal ideation with a specific plan and intent to act. The lifetime rate of suicide attempts was 40.8% (n = 106/260), among whom 35.9% (38/106) required medical hospitalization or intensive care. Of the 510 eligible subjects and 87 total deaths, the suicide (case fatality) rate was 3.53% (18/510), and the proportionate mortality rate was 20.6% (18/87).

The average number of attempts in the entire cohort was 1.34 (SD = 3.05, range 0–22). Among the suicide attempters, the mean number of attempts was 3.29 (SD = 4.10), and those who died by suicide had a similar average number of attempts (3.17, SD = 3.66) as the living participants (3.32, SD = 4.20). The suicide attempters made a total of 349 attempts, which means that one of every 19.4 attempters (349/18) completed suicide.

**Figure 2.**
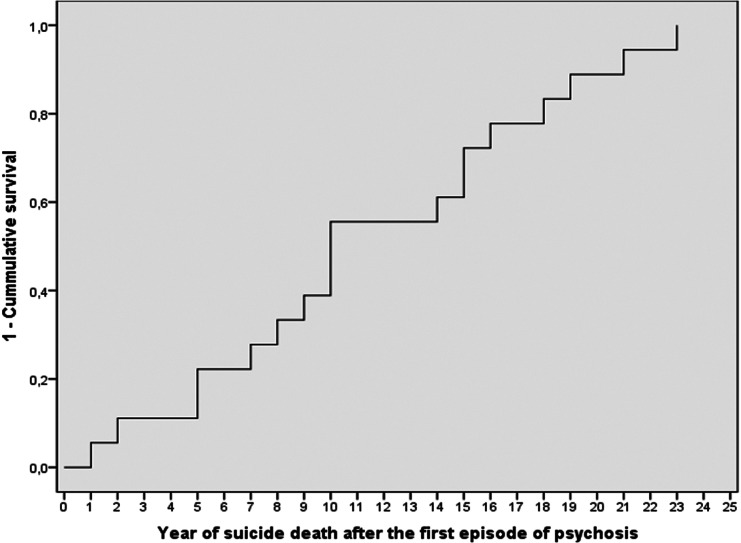
Kaplan–Meier analysis of the time to suicide over the follow-up period.

The Kaplan–Meier curve for the time the patients died by suicide after FEP increased steadily over the entire follow-up period (Figure 2). The mean time to suicide was 11.5 years (SD = 6.42, range = 1–23), and the median survival time was 10 years (interquartile range = 6.50–16.5). At the 5-year follow-up, 27.8% of the eventually suicidal patients had died by suicide, and at the 10-year follow-up, this figure was 55.6%.

### Associations among STB outcomes and among risk factors

The power analysis showed that the study’s sample size was adequate to conduct bivariate and multivariate analyses reliably (Supplementary Table S6). The severity of lifetime suicidal ideation was strongly correlated with both the number of suicide attempts (r = .481, *p* < 0.001) and lethality of the most severe attempt (r = .489, *p* < 0.001), while the correlation between the number of attempts and lethality was weaker (r = .235, *p* = 0.015).

Many putative risk factors were found to be significantly correlated. Of the 313 correlation coefficients, 150 (47.9%) were significant at *p* < 0.05, and 115 (36.7%) were significant at *p* < 0.01 (Supplementary Table S5). The most intercorrelated factors involved variables reflecting impaired development, from obstetric complications to adolescence factors, which showed weaker correlations with many other factors.

### Early predictors of STBs

Bivariate analyses showed that of the 26 putative risk factors, 14 were significantly correlated with suicidal thoughts severity, 17 with the number of suicide attempts, and 7 with the lethality of the most severe attempt (Table 2). Notably, neither illness onset nor FEP variables were significantly correlated with lethality of suicide attempts.

**Table 2.** Pearson’s correlation coefficients between predictor variables and suicidal thoughts and behaviors

	Suicidal thoughts (n = 260)	N. of suicide attempts (n = 260)	Lethality of suicide attempts (n = 106)
Sociodemographics:			
Age at follow-up or suicide	.008	.007	.048
Sex (female = 0, male = 1)	−.005	−.053	.236^a^
Years of follow-up	.130^a^	.148*	.126
Parental socioeconomic status	.144^a^	.105	−.054
Familial risk:			
Schizophrenia^†^	.077	.039	.022
Bipolar disorder^†^	−.063	−.034	−.101
Major depressive disorder^†^	.309^c^	.173^a^	.265^b^
Early developmental factors:			
Obstetric complications	.082	.143^a^	.242^a^
Neurodevelopmental delay	.175^b^	.121	.252^b^
Late developmental factors			
Childhood adversity	.224^c^	.201^b^	.215^a^
Premorbid adjustment	.118	.150*	.116
Social networks	.258^c^	.182^b^	.304^b^
Cognitive reserve	-.200^b^	-.201^b^	−.164
Precipitating factors:			
Psychosocial stressors	−.066	.048	-.231^a^
Drug abuse	−.010	−.081	.055
Illness-onset factors:			
Age at illness onset	−.116	-,078	-,034
DUP^†^	.056	.052	.014
DUCP^†^	.140^a^	.087	.045
First-episode characteristics:			
Mania	-.125^a^	.097	.038
Depression	.249^c^	.223^c^	−.024
Suicidal thoughts/attempts	.448^c^	.425^c^	.127
SAPS	−.057	−.069	−.066
SANS	.119	.080	−.006
Lack of insight	-.202^b^	-.142^a^	−.013
CGI illness severity	.160^a^	.159^a^	.132
CGI efficacy index	.153^a^	.069	.145

Abbreviations: DUP = Duration of Untreated Psychosis; DUCP = Duration of Untreated Continuous Psychosis NOS = Not Otherwise Specified; SAPS = Scale for the Assessment of Positive Symptoms; SANS = Scale for the Assessment of Negative Symptoms; CGI = Clinical Global Impression.

† Log-transformed scores

a = ^p^ < 0.05;

b = p < 0.01;

c = p < 0.001.

**Table 3.** Statistically significant unique associations between baseline predictors and lifetime suicidal thoughts and behaviours

Predictor variables	Suicidal thoughts (n = 260)					
	β (95% CI)	p	F change_(df)_	p	∆R^2^	Adj.R^2^
Parental socioeconomic status	.144 (.022 to .265)	0.021	5.43_(1,258)_	0.021	.021	.017
Familial load of MDD	.310 (.194 to .425)	<0.001	27.89_(1,257)_	<0.001	.096	.110
Neurodevelopmental delay	.130 (.013 to .246)	0.030	4.77_(1,256)_	0.030	.016	.123
Adolescence social networks	.215 (.1079 to .350)	<0.001	9.73_(1,255)_	0.002	.032	.151
Suicidal thoughts/attempts at FEP	.386 (.280 to .491)	<0.001	51.85_(1,255)_	<0.001	.142	.293

	Number of suicide attempts (n = 260)					
	β (95% CI)	p	F change_(df)_	p	∆R^2^	Adj. R^2^
Years of follow-up	.148 (.027 to .270)	0.017	5.80_(1,258)_	0.017	.022	.018
Familial load of MDD	.163 (.042 to .238)	0.008	7.09_(1,257)_	0.008	.026	.041
Obstetric complications	.125 (.005 to .245)	0.041	4.22_(1,256)_	0.041	.015	.053
Childhood adversity	.170 (.044 to .295)	0.008	7.07_(1,255)_	0.008	.025	.075
Suicidal thoughts/attempts at FEP	.382 (.270 to .494)	<0.001	45.44_(1,254)_	<0.001	.138	.212

	Lethality of suicide attempts (N = 106)					
	β (95% CI)	p	F change_(df)_	p	∆R^2^	Adj. R^2^
Sex (male)	.236 (.047 to .423)	0.015	6.13_(1,104)_	0.015	.056	.047
Familial load of MDD	.259 (.079 to .460)	0.006	7.85_(1,103)_	0.006	.067	.106
Neurodevelopmental delay	.239 (.054 to .384)	0.010	6.95_(1,102)_	0.016	.056	.154
Adolescence social networks	.212 (.009 to .413)	0.041	4.30_(1,101)_	0.041	.034	.181

Abbreviations: FEP = First-episode psychosis; MDD = Major depressive disorder

Multivariate analysis showed that the severity of suicidal thoughts was independently predicted by SES, familial load of MDD, neurodevelopmental delay, poor premorbid social networks, and suicidal thoughts/attempts at FEP. These factors explained 29.3% of the variance in suicidal thoughts, and the most predictive factor was suicidal thoughts/attempts at FEP, which uniquely explained 14.2% of the variance in the model (Table 3).

The number of suicide attempts was independently predicted by years of follow-up, familial load of MDD, obstetric complications, childhood adversity, and suicidal thoughts/attempts at FEP. These factors explained 21.2% of the variance in suicide attempts, and the most predictive factor was suicidal thoughts/attempts at FEP, which uniquely explained 13.8% of the variance in the model.

The lethality of the most severe attempt was independently predicted by male sex, the familial load of MDD, neurodevelopmental delay, and poor premorbid social networks. These factors explained 18.1% of the variance in lethality, and the most predictive factor was the familial load of MDD, which uniquely explained 6.7% of the variance in the model .

## Discussion

### Main findings

The current study traced STBs, such as their early determinants, in a large FEP cohort of 260 persons who were followed up over 21 years. This study has three key findings. First, we found that the lifetime rate of suicidal ideation was 62.7%, and 40.8% of the participants attempted suicide during follow-up. Eighteen patients died by suicide, representing 17.0% of suicide attempters, 3.53% of the initial sample (case fatality), and 20.6% of all deaths (proportionate mortality). The suicide rate after FEP increased steadily over the follow-up period, with a median survival time of 10 years.

Second, whereas bivariate analysis showed that many of the risk factors in several realms were associated with any of the suicidal outcomes, multivariate analysis showed that only a few risk factors independently predicted suicidality outcomes, which may be explained by substantial intercorrelation among risk factors, highlighting the relevance of multivariate adjustment in the prediction of STBs.

Third, different suicidality outcomes were largely predicted by different risk factors explaining between 18.1% and 29.3% of the outcomes. Key risk factors included familial load of MDD and some form of developmental impairment since they predicted each suicidality outcome. Suicidal thoughts/attempts at FEP were the most powerful predictors of lifetime suicide ideation and attempts. We also found that insight, depression, illness severity, and poor treatment response at FEP were associated with future STBs, albeit only in isolation of other risk factors.

Taken together, our findings outline the outstanding lifetime prevalence of STBs, such as their associated morbidity and mortality, over a very long-term course of psychotic disorders. Furthermore, our findings add to the literature in that they show that STBs can be independently predicted by a variety of early risk factors, which is of major relevance, given the long time lag between risk exposure and outcome assessment. Notwithstanding this, it should be acknowledged the multifaceted interplay between individual, interpersonal, and societal-level factors in shaping an individual’s vulnerability to suicide.

### Comparison with the literature

Our prevalence rates for suicidal ideation and attempts were much higher than those reported in the literature on psychosis (Sicotte et al., 2021), which may be explained by the longer follow-up period in this study. Our suicide mortality rate of 3.53% was in the middle of that estimated by the most recent review on the topic (Sicotte et al., 2021), and virtually the same rate (3.46%) was reported in a 20-year follow-up study of FEP (Starzer et al., 2023). A large, high-quality longitudinal FEP study with a mean follow-up of 11.5 years reported a case fatality from suicide of 1.9% and a proportionate mortality of 11.9%, with a median time to suicide of 5.6 years (Dutta et al., 2010). These figures are roughly half of those reported in our study, which may be because our follow-up length was twice that of Dutta et al.

In sharp contrast to our steady increase in suicide rates over follow-up, previous studies have suggested that suicide rates show a downward trend over time (Palmer et al., 2005; Pompili et al., 2011; Ran et al., 2020), although a recent meta-analysis showed that this trend was not statistically significant between 0–10 years and 11–20 years (Fu et al., 2023). This discrepancy may be partly due to the short duration of most previous studies, which was typically less than 10 years. Longer studies have shown that suicide risk persists for a decade after FEP (Lindelius & Kay, 1973; Dutta et al., 2010). Similarly, a meta-analysis found that there was a numerically but not statistically significant greater suicide-related mortality among incident versus prevalent cases of schizophrenia (Correll et al., 2022).

Overall, our findings confirm the most agreed upon early risk factors for later STBs in FEP, namely male sex, depressive symptoms, and suicidal ideation/attempts (Sicotte et al., 2021). However, we found that depression was no longer significant in multivariate analysis. To the best of our knowledge, our study is the first to establish a strong link between the familial load of MDD and different types of STBs in people with psychotic disorders, which aligns with family studies conducted in nonpsychotic populations (Astruc et al., 2004; van Dijk, Murphy, Posner, Talati, & Weissman, 2021; Weissman et al., 2016).

A novel finding of our study was that several indicators of impaired development were associated with lifetime STBs in both bivariate and multivariate analyses. This finding can be framed within the relevance of impaired neurodevelopment in psychotic disorders (Murray, Bhavsar, Tripoli, & Howes, 2017), which makes individuals more vulnerable to a range of poor outcomes, including STBs. No previous study has examined the link between developmental factors and later STBs in individuals with psychotic disorders, except for premorbid adjustment (with inconclusive findings) (Sicotte et al., 2021) and childhood adversity (with strong evidence) (Baldini et al., 2023). However, several studies on the developmental predictors of STBs in nonpsychotic populations have provided evidence for such a link. A recent meta-analysis of birth cohort studies supported an association between many unfolding developmental factors, including obstetric complications, and subsequent suicide behaviors (Vidal-Ribas et al., 2022). Another study showed that normal siblings of individuals with neurodevelopmental conditions have four times the risk of suicide attempts compared to controls (Wolff, Franco, Magiati, Pestell, & Glasson, 2023). This study also showed that a history of cognitive dysfunction, childhood adversity, depression, and psychosis (among other factors) had the highest relative importance in predicting lifetime suicidality among siblings. Finally, a study of children and adolescents admitted for a serious suicide attempt showed that 70% had neurodevelopmental deviance traits (Lévy-Bencheton, Chaste, & Sansen, 2024).

We observed that the risk factors were strongly interrelated, and according to the timing of exposure, it appears that prenatal factors, together with perinatal factors, lead to a cascade of interconnected developmental factors from infancy to childhood and adolescence. The mechanism underlying the association between developmental impairment and STBs is complex and multifactorial, although it has been speculated that impaired development influences the immediate context in which the child is raised by increasing the likelihood of being exposed to social and psychological factors conferring vulnerability to suicidal behaviors (Vidal-Ribas et al., 2022).

No previous study specifically examined obstetric complications, neurodevelopmental delay, premorbid cognitive reserve, premorbid social networks, DUCP, illness severity, and early response to treatment as risk factors of STBs in psychosis; thus, our findings regarding these variables should be confirmed by future long-term studies.

### Clinical implications

Our findings may help clinicians improve the prediction and prevention of STBs in subjects with psychotic disorders in several ways. First, it is essential to acknowledge that experiencing a psychotic disorder conveys a higher risk of STBs than that observed in normal people and most other mental disorders, and that this risk is present over the entire illness course.

Second, while we identified key independent risk factors for later STBs, we also observed that many other factors, ranging from prenatal factors to FEP characteristics, were not independently associated with STBs. Our findings should encourage clinicians to systematically assess all of these risk factors to detect and treat at-risk individuals as early as possible. In the era of personalized medicine, our findings suggest that a simple assessment of a personal history of developmental impairment, depression, and suicidal thoughts and attempts, such as a positive family history of MDD, as part of the routine clinical assessment of people with FEP, can help predict the long-term risk of suicidal behaviors.

Third, although preventive actions should ideally be directed toward each risk factor, a more practical option is to focus interventions on the most consistent and preventable factors. Most risk factors for STBs predate illness onset; therefore, they are not amenable to preventive interventions in established psychosis. In this regard, interventions that target depression and suicidal thoughts in people with FEP may help address more efficiently later STBs. In connection, close monitoring of these variables over the course of illness, along with contextual factors, appears to be the most appropriate way to prevent STBs.

### Limitations

Our findings should be interpreted within the context of several limitations. First, we used an epidemiologically based first-admission sample, which may be biased toward more severe clinical outcomes than epidemiological incident samples.

Second, we had a relatively high dropout rate (50.9%), which, although typical for very long-term designs, limits our ability to generalize the findings to patients lost to follow-up. We did not find evidence of selection bias regarding baseline variables between participants and nonparticipants, including mood symptoms and suicidal thoughts/attempts at FEP, suggesting that attrition had a minimal impact on the results.

Third, risk factors other than FEP characteristics were assessed retrospectively at the time of FEP, which are subject to misreporting due to recall bias. We sought to mitigate this issue by integrating participant data with information from family members as well as medical and social records.

Fourth, although we assessed many relevant predictor variables, they were not fully comprehensive, and data on some important risk factors, such as premorbid STBs and a family history of suicide attempts, were not available.

Fifth, given the primary exploratory nature of our study, we did not correct for multiple comparisons; thus, some false-positive findings were possible. However, it should be noted that analyses were performed hierarchically from bivariate to multivariate, which decreased the probability of type I error.

Finally, the number of those who died by suicide within the study was low (n = 18), which precluded us from specifically examining its baseline predictors owing to a lack of statistical power.

## Conclusions

In summary, our findings suggest that people with FEP are highly vulnerable to STBs over a long-term illness course. Overall, it is suggested that people with FEP and a first-degree relative with MDD, antecedents of neurodevelopmental deviance in several realms, and suicidal thoughts or attempts at FEP should be carefully monitored for future suicide behaviors. Our findings should encourage clinicians to systematically assess all these risk factors to detect and treat individuals at high risk of suicide as early as possible and over the course of illness.

## Supporting information

Peralta et al. supplementary material Peralta et al. supplementary material

## Supplementary material

The supplementary material for this article can be found at http://doi.org/10.1017/S0033291725000443.
