# Supplementary material for: Lifetime suicidal thoughts, attempts, and lethality of attempts as major outcome domains of psychotic disorders: a 21-year prospective cohort study after a first-episode psychosis
Source: Psychol Med. 2025 Mar 4;55:e69. doi: 10.1017/S0033291725000443 (PMC12055022; doi:10.1017/S0033291725000443)
Supplement: Peralta et al. supplementary material [file S0033291725000443sup001.docx]

**Supplementary material**

Supplementary Methods

Supplementary Table S1. Causes of nonsuicide mortality according the Spanish National Institute for Statistics classification (N=69)

Supplementary Table S2. Distribution of sex, age at follow-up or suicide, number of suicide attempts, and DSM-5 diagnosis among the living participants (n=243) and among those who died of suicide (n=18).

Supplementary Table S3. Frequency distribution of lifetime most severe suicidal ideation (n=260).

Supplementary Table S4. Frequency distribution of number of suicide attempts in the living participants and in those who died of suicide.

Supplementary Table S5. Frequency distribution of actual lethality/medical damage in those participants with one or more suicide attempts (n=106).

Supplementary Table S6. Power analysis for required sample size, given α=0.05, power=0.80 and medium effect size, such as post-hoc computed achieved power given α=0.05, actual sample size and medium effect size.

Supplementary Table S7. Pearson’s correlation coefficients among predictor variables.

**Supplementary Methods**

**Cognitive reserve assessment.**

Premorbid intelligence quotient (IQ), educational attainment level, and lifetime participation in leisure, social, and physical activities are the three most commonly proposed proxy indicators of cognitive reserve in psychiatry, particularly in first-episode psychosis (Amoretti et al., 2018) and were used to assess cognitive reserve in this study.

Estimated premorbid IQ was evaluated with the Word Accentuation Test (WAT), which is the Spanish equivalent of the National Adult Reading Test. We used the WAIS III full scale IQ equivalence of the WAT scores as reported by Gomar et al. (2011) to obtain the premorbid IQ score as a measure of crystallized intelligence.

The total number of participants’ completed years in education, was used to assess educational attainment level.

The scholastic performance domain of the Premorbid Assessment Scale, as included in the Comprehensive Assessment of Symptoms and History, was used to evaluate adolescence participation in leisure, social, and physical activities and by enquiring about involvement in social activities, their self-rated capacity to take part in physical activities and satisfaction with hobbies.

A “Cognitive Reserve Score” was created via a Principal Components Analysis (PCA) for each subject for the three core proxy indicators, with higher scores indicating better performance.

References:

Amoretti S, Cabrera B, Torrent C, Mezquida G, Lobo A, González-Pinto A, et al. Cognitive reserve as an outcome predictor: first-episode affective versus non-affective psychosis.Acta Psychiatr Scand. 2018;138(5):441–55

Gomar JJ, Ortiz-Gil J, McKenna PJ, et al. Validation of the Word Accentuation Test

(TAP) as a means of estimating premorbid IQ in Spanish speakers. *Schizophr Res* May

2011;128(1-3):175-176.

**Columbia-Suicide Severity Rating Scale (CSSRS)**

The lifetime version of the CSSRS assesses various aspects of suicidal thoughts and behaviours across the entire life span using an array of questions designed to evaluate the frequency and severity of ideation and behavior. The questions contained in the scale are suggested probes, and ultimately, the determination of the presence of suicidal ideation or behavior depends on the judgment of the clinician individual administering the scale.

The questionnaire includes queries on several aspects of suicidal ideation severity, from wishes to be dead to active suicidal ideation with a specific plan and intent. The rater must score the lifetime most severe suicide ideation.

Additionally, the questionnaire incorporates queries on suicidal behavior, including the number of suicidal attempts, defined as potentially self-injurious act undertaken with at least some wish to die, because of act. The rater must score the total number of suicidal attempts.

In cases where a patient has made actual attempts, the questionnaire asks about the lethality or medical damage of the most severe attempt.

Suicidal ideation and lethality are each rated on a 6-point Likert scale (see Supplementary Tables S3 and S5 for the definition of specific anchor points of each scale).

Reference:

Posner, K., Brent, D., Lucas, C., Gould, M., Stanley, B., Brown, G., … Mann, J. (2008). *Columbia-Suicide Severity Rating Scale (C-SSRS)*. The Research Foundation for Mental Hygiene, Inc.

Supplementary Table S1. Causes of nonsuicide mortality according the Spanish National Institute for Statistics classification (N=69)

|  | N | % |
| --- | --- | --- |
| Multiple causes | 16 | 23.1 |
| Cancer | 12 | 17.4 |
| Respiratory diseases | 9 | 13.0 |
| Cardio-vascular system diseases | 8 | 11.6 |
| Infectious diseases | 7 | 10.1 |
| Accidents | 7 | 10.1 |
| Unknown | 6 | 8.7 |
| Other causes | 4 | 5.8 |

Supplementary Table S2. Distribution of sex, age at follow-up or suicide, number of suicide attempts, and DSM-5 diagnosis among the living participants (n=243) and among those who died by suicide (n=18)

|  | Living participants | Died by suicide | X^2^ or t_(df)_ | p |
| --- | --- | --- | --- | --- |
| Sex, male, n (%) | 137 (56.6) | 14 (77.8) | 2..275_(1)_ | 0.069* |
| Age at follow-up or suicide, y | 48.5 (10.4) | 40.0 (9.43) | 3.365_(258)_ | 0.001 |
| Number of suicide attempts | 1.21 (2.99) | 3.17 (3.66) | 2.639_(258)_ | 0.009 |
| DSM-5 diagnosis, n (%): |  |  |  |  |
| Schizophrenia | 113 (46.7) | 9 (50.0) | 6.421_(7)_ | 0.492 |
| Schizophreniform disorder | 6 (2.5) | 0 |  |  |
| Brief psychotic disorder | 20 (8.3) | 3 (13.0) |  |  |
| Delusional disorder | 4 (1.7) | 0 |  |  |
| Schizoaffective disorder | 38 (15.7) | 5 (27.7) |  |  |
| Bipolar disorder | 42 (17.4) | 1 (2.3) |  |  |
| Major depressive disorder | 10 (4.1) | 0 |  |  |
| Psychotic disorder NOS | 9 (3.7) | 0 |  |  |

*Fisher exact text

DSM-5= Diagnostic and Statistical Manual, 5^th^ version; NOS= not otherwise specified.

Notes:

One patient that was successfully followed and died by suicide short after the follow-up assessment was included in the suicide group.

The diagnosis of suicide patients was the last known diagnosis before suicide as recorded in clinical registers.

Supplementary Table S3. Frequency distribution of lifetime most severe suicidal ideation (n=260)

|  | N | % |
| --- | --- | --- |
| 1. Lack of suicidal thoughts | 73 | 28.1 |
| 1. Wish to be dead | 24 | 9.2 |
| 2. Non-specific active suicidal thoughts | 30 | 11.5 |
| 3. Active suicidal ideation with any methods (not plan)  without intent to act | 20 | 7.7 |
| 4. Active suicidal ideation with some intent to act, without  specific plan | 23 | 8.8 |
| 5. Active suicidal ideation with specific plan and intent | 90 | 34.6 |

Supplementary Table S4. Frequency distribution of number of suicide attempts in the living participants and in those who died of suicide.

| Number of suicidal attempts | Living participants (n=243) | | Death by suicide (n=18) | | Total  (n=260) | |
| --- | --- | --- | --- | --- | --- | --- |
|  | N | % | N | % | N | % |
| 0 | 154 | 63,4 | ‒ | ‒ | 154 | 59,2 |
| 1 | 39 | 16,0 | 5 | 27.8 | 44 | 16,9 |
| 2 | 16 | 6,6 | 7 | 38.9 | 22 | 8,5 |
| 3 | 11 | 4,5 | 4 | 22.2 | 15 | 5,8 |
| 4 | 7 | 2,9 | ‒ | ‒ | 7 | 2,7 |
| 5 | 5 | 2,1 | ‒ | ‒ | 5 | 1,9 |
| >5 | 11 | 4.5 | 2 | 11.2 | 13 | 5.0 |
| Mean (SD) [range] | 1.21 (2.98) [0‒22] | | 3.17 (3.66) [1‒14] | | 1.34 (3.05) [0‒22] | |

Supplementary Table S5. Frequency distribution of actual lethality/medical damage in those participants with one or more suicide attempts (n=106)

|  | N | % |
| --- | --- | --- |
| 0. No physical damage or very minor physical damage | 4 | 3.8 |
| 1. Minor physical damage | 20 | 18.9 |
| 2. Moderate physical damage; medical attention needed | 26 | 24.5 |
| 3. Moderately severe physical damage; medical  hospitalization and likely intensive care required | 22 | 20.8 |
| 4. Severe physical damage; medical hospitalization with  intensive care required | 16 | 15.1 |
| 5. Death | 18 | 17.0 |
| Mean (SD) [range] | 2.75 (1.46) [0‒5] | |

Supplementary Table S6. Power analysis for required sample size, given α=0.05, power=0.80 and medium effect size, such as post-hoc computed achieved power given α=0.05, actual sample size and medium effect size

| Bivariate analysis | Required sample size | Actual sample size | Achieved power |
| --- | --- | --- | --- |
|  | 85 | 260 | 0.99 |
|  | 85 | 106^†^ | 0.88 |

,

| Multiple regression | Required sample size | Actual sample size | Achieved power |
| --- | --- | --- | --- |
| N. of predictors=17 | 72 | 260 | 1.00 |
| N. of predictors=14 | 66 | 260 | 1.00 |
| N. of predictors=7 | 51 | 106^†^ | 0.99 |

^†^ This sample size corresponds to the variable lethality of the most severe suicide attempt, which was only assessed in subjects with al least one suicide attempt.

Reference:

Faul F, Erdfelder E, Lang AG, Buchner A. G*Power 3: A flexible statistical power analysis program for the social, behavioral, and biomedical sciences. Behav Res Methods. 2007 May;39(2):175-91. doi: 10.3758/bf03193146. PMID: 17695343.

Supplementary Table S7. Pearson’s correlation coefficients among predictor variables

|  | 1 | 2 | 3 | 4 | 5 | 6 | 7 | 8 | 9 | 10 | 11 | 12 | 13 | 14 | 15 | 16 | 17 | 18 | 19 | 20 | 21 | 22 | 23 | 24 | 25 |
| --- | --- | --- | --- | --- | --- | --- | --- | --- | --- | --- | --- | --- | --- | --- | --- | --- | --- | --- | --- | --- | --- | --- | --- | --- | --- |
| 1.Age at follow-up or suicide | * |  |  |  |  |  |  |  |  |  |  |  |  |  |  |  |  |  |  |  |  |  |  |  |  |
| 2.Sex (female=0, male=1) | **-.16** | * |  |  |  |  |  |  |  |  |  |  |  |  |  |  |  |  |  |  |  |  |  |  |  |
| 3.Years of follow-up/until suicide | **.42** | -.01 | * |  |  |  |  |  |  |  |  |  |  |  |  |  |  |  |  |  |  |  |  |  |  |
| 4.Parental socioeconomic status | **.25** | .09 | **.25** | * |  |  |  |  |  |  |  |  |  |  |  |  |  |  |  |  |  |  |  |  |  |
| 5. Familial risk of SSD^†^ | -.04 | .03 | **.15** | .07 | * |  |  |  |  |  |  |  |  |  |  |  |  |  |  |  |  |  |  |  |  |
| 6.Familial risk of BD^†^ | **-.28** | -.02 | **-.13** | -.09 | .07 | * |  |  |  |  |  |  |  |  |  |  |  |  |  |  |  |  |  |  |  |
| 7.Familial risk of MDD^†^ | -.08 | **.13** | -.04 | -.01 | -.03 | -.04 | * |  |  |  |  |  |  |  |  |  |  |  |  |  |  |  |  |  |  |
| 8.Obstetric complications | -.06 | .06 | .06 | .08 | **.16** | .09 | .04 | * |  |  |  |  |  |  |  |  |  |  |  |  |  |  |  |  |  |
| 9.Neurodevelopmental delay | **-.19** | **.13** | .07 | **.18** | **.27** | .00 | .08 | **.66** | * |  |  |  |  |  |  |  |  |  |  |  |  |  |  |  |  |
| 10.Childhood adversity | -.07 | .00 | .00 | .11 | **.15** | .00 | .01 | **.34** | **.52** | * |  |  |  |  |  |  |  |  |  |  |  |  |  |  |  |
| 11.Premorbid adjustment | -.10 | .04 | -.01 | **.15** | **.27** | .06 | -.09 | **.47** | **.59** | **.66** | * |  |  |  |  |  |  |  |  |  |  |  |  |  |  |
| 12.Social networks | -.08 | **.16** | -.03 | **.16** | **.21** | -.04 | .06 | **.43** | **.55** | **.63** | **.72** | * |  |  |  |  |  |  |  |  |  |  |  |  |  |
| 13.Cognitive reserve | -.04 | -.11 | **-.13** | **.17** | **-.20** | .03 | -.01 | **-.40** | **-.62** | **-.54** | **-.69** | **-.55** | * |  |  |  |  |  |  |  |  |  |  |  |  |
| 14.Psychosocial stressors | .08 | **-.25** | -.01 | -.06 | **-.18** | -.08 | -.02 | **-.16** | **-.23** | **-.23** | **-.17** | **-.23** | **.20** | * |  |  |  |  |  |  |  |  |  |  |  |
| 15.Drug abuse | **-.20** | **.21** | **-.16** | -.07 | .00 | .06 | -.11 | -.07 | .04 | **.14** | .06 | .05 | -.09 | **-.22** | * |  |  |  |  |  |  |  |  |  |  |
| 16. Age at illness onset | **.46** | **-.14** | **-.14** | .08 | -.12 | **.23** | -.06 | **-.15** | **-.31** | **-.19** | **-.19** | **-.15** | .12 | .10 | -.11 | * |  |  |  |  |  |  |  |  |  |
| 17.DUP^†^ | .00 | .04 | **-.15** | .01 | .03 | .02 | -.02 | **.24** | **.27** | **.42** | **.39** | **.41** | **-.25** | **-.17** | .03 | -.11 | * |  |  |  |  |  |  |  |  |
| 18.DUCP^†^ | .08 | .04 | -.08 | .06 | .06 | -.01 | -.03 | **.28** | **.37** | **.51** | **.47** | **.53** | **-.36** | **-.23** | .04 | -.12 | **.85** | * |  |  |  |  |  |  |  |
| 19.Manic symptoms | -.01 | .03 | -.02 | -.08 | -.05 | .12 | .07 | -.10 | **-.16** | **-.21** | **-.28** | **-.30** | **.17** | -.04 | .07 | .03 | **-.22** | **-.24** | * |  |  |  |  |  |  |
| 20.Depressive symptoms | .05 | -.11 | .03 | .03 | -.06 | .01 | **.15** | -.03 | -.08 | -.02 | .02 | -.01 | .01 | **.23** | **-.22** | .04 | .01 | -.01 | **-.18** | * |  |  |  |  |  |
| 21.Suicidal thoughts/attempts | -.04 | .07 | .07 | .04 | .03 | -.12 | .12 | .05 | .08 | **.18** | **.15** | **.18** | **-.13** | .07 | -.09 | -.11 | **.13** | .11 | **-.22** | **.48** | * |  |  |  |  |
| 22.SAPS | **-.19** | .02 | -.10 | -.09 | .05 | .00 | .00 | -.04 | .01 | .11 | .00 | .03 | -.05 | **-.13** | **.17** | **-.18** | .08 | .11 | **.18** | **-.40** | **-.13** | * |  |  |  |
| 23.SANS | -.08 | .03 | .09 | .11 | .01 | .01 | .02 | **.25** | **.33** | **.47** | **.46** | **.48** | **-.33** | -.01 | -.02 | **-.19** | **.32** | **.37** | **-.35** | **.39** | **-.26** | -.05 | * |  |  |
| 24.Lack of insight | **-.31** | .09 | .07 | -.07 | .08 | .04 | -.06 | -.01 | .03 | .03 | -.06 | -.03 | .02 | **-.21** | .20 | **-.13** | .02 | .05 | **.48** | **-.67** | **-.32** | **.67** | **-.30** | * |  |
| 25.CGI- illness severity | -.08 | .07 | -.05 | .08 | **.16** | .03 | .01 | **.32** | **.44** | **.73** | **.69** | **.65** | **-.46** | **-.26** | **.13** | **-.19** | **.59** | **.66** | **-.22** | -.02 | **.15** | .08 | **.43** | .03 | * |
| 26.CGI- efficacy index | -.06 | .05 | .04 | .05 | **.14** | -.06 | -.02 | **.23** | **.37** | **.63** | **.51** | **.57** | **-.32** | **-.18** | .05 | **-.21** | **.43** | **.54** | **-.20** | .01 | **.15** | .03 | **.46** | .01 | **.70** |

r values ±.13, .17, and .21 are statistically significant at p values of <0.05, <0.01, and <0.001, respectively.

BD= Bipolar disorder; CGI= Clinical Global Impression; DUP= Duration of Untreated Psychosis; DUCP= Duration of Untreated Continuous Psychosis; MDD= Major Depressive Disorder; SAPS= Scale for the Assessment of Positive Symptoms; SANS= Scale for the Assessment of Negative Symptoms; SSD= Schizophrenia Spectrum Disorders.
